# Influenza polymerase encoding mRNAs utilize atypical mRNA nuclear export

**DOI:** 10.1186/1743-422X-11-154

**Published:** 2014-08-28

**Authors:** Sean Larsen, Steven Bui, Veronica Perez, Adeba Mohammad, Hilario Medina-Ramirez, Laura L Newcomb

**Affiliations:** Department of Biology, California State University San Bernardino, 5500 University Parkway, San Bernardino, CA 92407 USA

**Keywords:** Influenza, Virus, mRNA, Nuclear export

## Abstract

**Background:**

Influenza is a segmented negative strand RNA virus. Each RNA segment is encapsulated by influenza nucleoprotein and bound by the viral RNA dependent RNA polymerase (RdRP) to form viral ribonucleoproteins responsible for RNA synthesis in the nucleus of the host cell. Influenza transcription results in spliced mRNAs (M2 and NS2), intron-containing mRNAs (M1 and NS1), and intron-less mRNAs (HA, NA, NP, PB1, PB2, and PA), all of which undergo nuclear export into the cytoplasm for translation. Most cellular mRNA nuclear export is Nxf1-mediated, while select mRNAs utilize Crm1.

**Methods:**

Here we inhibited Nxf1 and Crm1 nuclear export prior to infection with influenza A/Udorn/307/1972(H3N2) virus and analyzed influenza intron-less mRNAs using cellular fractionation and reverse transcription - quantitative polymerase chain reaction (RT-qPCR). We examined direct interaction between Nxf1 and influenza intron-less mRNAs using immuno purification of Nxf1 and RT-PCR of associated RNA.

**Results:**

Inhibition of Nxf1 resulted in less influenza intron-less mRNA export into the cytoplasm for HA and NA influenza mRNAs in both human embryonic kidney cell line (293 T) and human lung adenocarcinoma epithelial cell line (A549). However, in 293 T cells no change was observed for mRNAs encoding the components of the viral ribonucleoproteins; NP, PA, PB1, and PB2, while in A549 cells, only PA, PB1, and PB2 mRNAs, encoding the RdRP, remained unaffected; NP mRNA was reduced in the cytoplasm. In A549 cells NP, NA, HA, mRNAs were found associated with Nxf1 but PA, PB1, and PB2 mRNAs were not. Crm1 inhibition also resulted in no significant difference in PA, PB1, and PB2 mRNA nuclear export.

**Conclusions:**

These results further confirm Nxf1-mediated nuclear export is functional during the influenza life cycle and hijacked for select influenza mRNA nuclear export. We reveal a cell type difference for Nxf1-mediated nuclear export of influenza NP mRNA, a reminder that cell type can influence molecular mechanisms. Importantly, we conclude that in both A549 and 293 T cells, PA, PB1, and PB2 mRNA nuclear export is Nxf1 and Crm1 independent. Our data support the hypothesis that PA, PB1, and PB2 mRNAs, encoding the influenza RdRP, utilize atypical mRNA nuclear export.

## Background

Influenza A virus remains a health menace and while yearly statistics vary, ~36,000 deaths and ~220,000 hospitalizations are attributed to influenza each year (mean value) in the United States [1]. Moreover, the rapidly evolving nature of this segmented RNA virus leads to the emergence of new, unseen subtypes, which have potential to cause a pandemic. Influenza pandemics have occurred at three times in the last century (1918, 1957 and 1968) and once in the current century (2009). Fortunately, the 2009 novel H1N1 influenza virus proved not as pathogenic as initially expected, resulting in fewer deaths than predicted. However, the high transmissibility saw the novel H1N1 readily sweep the globe and resulted in a high global economic burden [2]. Findings that the highly pathogenic H5N1 avian influenza is able to evolve increased transmissibility in the ferret model [3, 4], has again emphasized the real possibility that a mutation or recombination event could result in the emergence of a highly pathogenic and easily transmissible influenza virus that will cause a severe and deadly pandemic. Greater understanding of the molecular mechanisms of influenza replication will facilitate the ability to identify novel antiviral targets and develop effective antiviral therapies.

Influenza is a segmented negative strand RNA virus. Each RNA segment is encapsulated by influenza nucleoprotein (NP) and bound by the viral RNA dependent RNA polymerase (RdRP) to form viral ribonucleoproteins (vRNPs) responsible for RNA transcription. Unlike most negative strand RNA viruses, which transcribe RNA in the cytoplasm, influenza transcribes its mRNA in the nucleus. Transcription is primed with a capped 12–15 nucleotide RNA excised from nascent cellular mRNAs by the RdRP in a process termed “cap-snatching” [5] and polyadenylated by reiterative copying at a poly U stretch near the 5’ end of the vRNA template [6]. Two viral transcripts, M and NS, are spliced to generate alternative mRNAs. Virus replication requires the nuclear export of spliced mRNAs (M2 and NS2), intron-containing mRNAs (M1 and NS1), and intron-less mRNAs (HA, NA, NP, PB1, PB2, and PA).

The major structure involved in nuclear trafficking is an assembly of nucleoporins located within the nuclear envelope termed the Nuclear Pore Complex (NPC). Large molecular weight complexes require nuclear export signals to export through the NPC. RNAs are transported by interaction with proteins. Studies of retroviral mRNA nuclear export have led to our understanding of two distinct host mRNA nuclear export pathways, represented by the proteins Crm1 and Nxf1. Complex retroviruses such as HIV encode viral Rev protein, which binds the Rev-responsive element (RRE) found within the introns of intron-containing completely un-spliced and partially spliced viral mRNAs, and assists in the export of these viral mRNAs from the nucleus [7]. Rev contains the first described nuclear export signal (NES) which interacts with host Crm1 to export Rev, along with the bound viral intron containing mRNA, to the cytoplasm [8]. Simple retroviruses such as Mason-Pfizer monkey virus (MPMV) encode an RNA structure within introns termed the constitutive transport element (CTE), which was used to identify host **n**uclear e**x**port **f**actor 1 (Nxf1, also called TAP) as a cellular mRNA nuclear export factor [9]. Over-expression of this RNA element blocks most cellular mRNA nuclear export but not Rev dependent mRNA nuclear export [10]. Conversely, a mutant nucleoporin, which inhibits Crm1, blocks Rev dependent RNA export but not bulk cellular mRNA export or CTE dependent export [11]. Therefore, the two host cellular proteins, Crm1 and Nxf1, represent two separate mRNA nuclear export pathways.

Research on the mechanisms of influenza mRNA nuclear export is insufficient and results contradictory. RNAi screening in *drosophila* cells identified Nxf1 as an essential host factor for influenza mRNA nuclear export [12]. Additional studies provide evidence of a role for host Nxf1 in export of some but not all influenza mRNAs [13, 14]. In contrast, another report concludes that influenza NS1 protein inhibits host Nxf1 nuclear export to block expression of host antiviral mRNAs such as IFN mRNAs [15]. The latter paper suggests influenza mRNA nuclear export is not Nxf1-mediated, but rather Crm1-mediated. While Crm1 nuclear export is utilized by influenza virus for export of viral ribonucleoproteins (vRNPs) during virion assembly [16], reports support host Crm1 is not used by any influenza mRNAs for export from the nucleus [13, 14, 17, 18]. The published studies were performed in kidney cells, either Madin-Darby canine kidney cell line (MDCK), baby hamster kidney cell line (BHK), and/or human embryonic kidney cell line (293 T). Given that influenza virus infects cells of the respiratory tract, human lung adenocarcinoma epithelial cell line (A549) are likely a better model cell line for studies of influenza infection. Therefore, we set out to examine influenza viral mRNA export in human lung adenocarcinoma epithelial cell line (A549).

Here we report our results on the role of Nxf1 and Crm1 in influenza intron-less mRNA nuclear export (HA, NA, NP, PB1, PB2, and PA mRNAs). We utilized both inhibition of Nxf1 or Crm1 and direct immuno purification of Nxf1 along with associated RNAs. We find influenza mRNA nuclear export is Nxf1-mediated with the exception of the influenza RNA dependent RNA polymerase encoding mRNAs; PA, PB1, and PB2. Our results in A549 cells differed from our results and published research obtained in 293 T cells [13] with respect to the export of influenza NP mRNA. This led us to conclude there is a cell type difference in Nxf1-mediated NP mRNA nuclear export: in human lung adenocarcinoma epithelial cell line (A549) NP mRNA nuclear export is Nxf1-mediated while in human embryonic kidney cell line (293 T) NP mRNA nuclear export is Nxf1 independent. It is important to acknowledge cell type differences if the larger goal is to translate data to application.

Although much research suggests Crm1 is not utilized for influenza mRNA nuclear export [13, 14, 17, 18], in light of the revelation of a cell type difference, we readdressed the role of Crm1 in influenza mRNA nuclear export in A549 cells. Inhibition of Crm1 did not result in significant inhibition of nuclear export of any influenza mRNAs analyzed. This led us to conclude that the influenza RNA dependent RNA polymerase encoding mRNAs; PA, PB1, and PB2, do not export the nucleus via the two defined mRNA nuclear export pathways represented by Crm1 and Nxf1, but instead use an atypical mRNA nuclear export pathway. Defining this pathway will shed light on alternate cellular mRNA nuclear export pathways and may lead to the discovery of novel antiviral targets.

## Results and discussion

### Inhibition of Nxf1-mediated nuclear export via expression of dominant negative Nxf1 results in decreased virus production and cytoplasmic reduction of select influenza mRNAs

**Figure 1:**
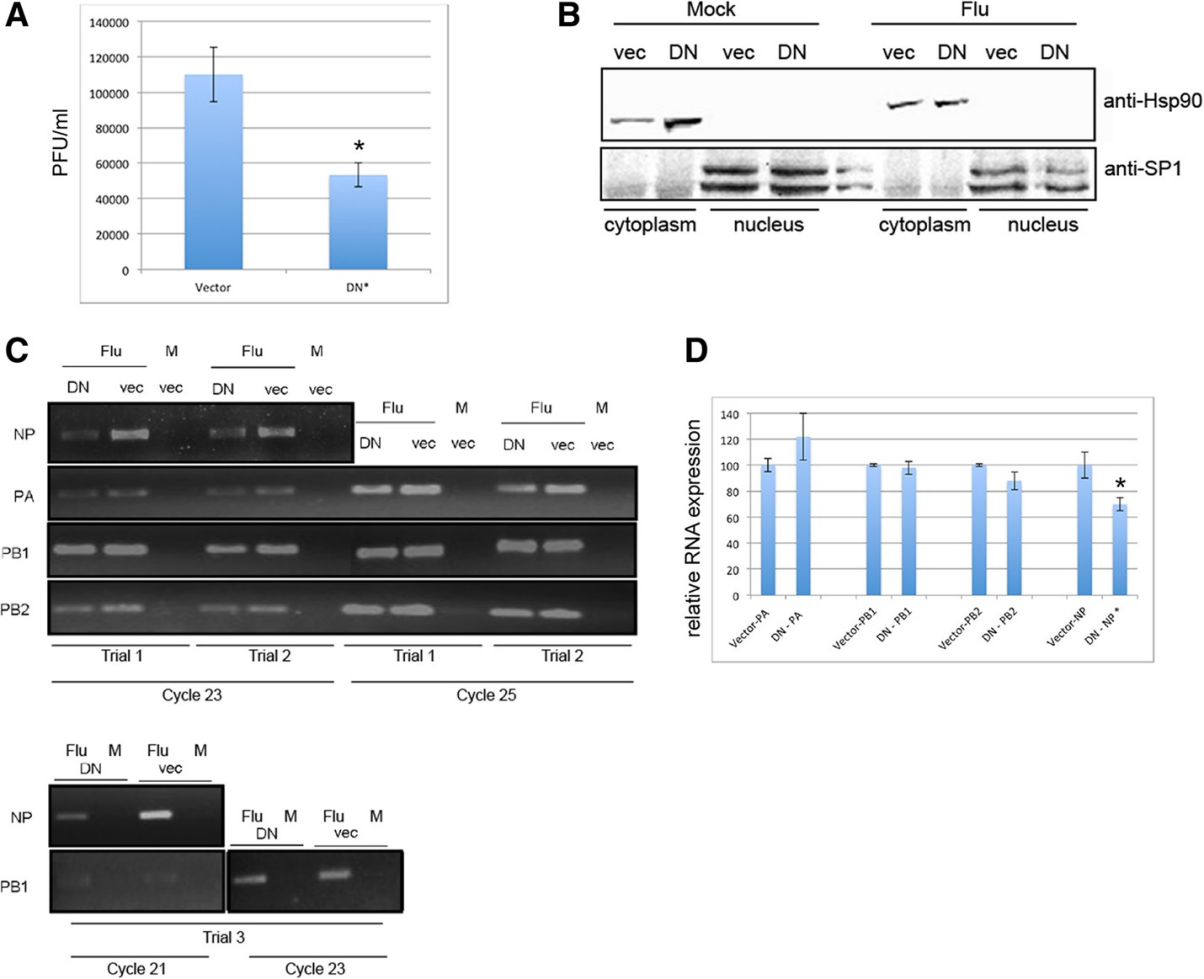
**Expression of dominant negative Nxf1 decreases virus production and results in cytoplasmic reduction of select influenza mRNAs.** A549 cells were transfected with plasmid to express dominant negative Nxf1 (DN) or vector control (vec) and infected with influenza A Udorn at 2.5 MOI 48 hours post transfection. **A**. Virus production at 12 hours post infection with influenza A Udorn. An asterisk indicates statistical difference between cells transfected with DN-Nxf1 compared to vec-control. Data presented is from biological triplicate trials. **B**. Cells transfected with DN-Nxf1 or vector for 48 hours and subsequently infected with influenza A Udorn at 2.5 MOI for 3.5 hours were fractionated. Cytoplasm and nuclear protein fractions were separated by SDS-PAGE and subject to Western blot to detect SP1 and Hsp90. Shown is a representative blot from one biological trial. **C**. RT-semi-qPCR of RNA isolated 3.5 hours post infection from the cytoplasm fraction. RNA was quantified and equal concentrations subject to RT with oligo dT. Gene specific PCR was performed using primers to amplify NP, PA, PB1 and PB2 as indicated. Data show sequential PCR cycles from three biological independent trials. **D**. RT-qPCR of RNA isolated from one of the above biological trials performed in triplicate. Delta Ct was calculated to determine relative RNA expression. Raw CT values were analyzed in Microsoft Excel using 2^ΔCt(average control- average treated)^. Standard error was obtained by calculating the standard deviation of the sample set divided by the square root of the sample set size, and indicated using error bars. Significance was determined using a two-tailed T-Test conducted in Microsoft Excel, and judging any p value less than .05 as significant, indicated by an asterisk.

We inhibited Nxf1-mediated nuclear export by expression of a dominant negative Nxf1 protein encoding alanine substitutions at residues 593–595 within the nuclear export signal (NES), rendering a dominant negative effect on Nxf1-mediated nuclear export [19]. We expressed dominant negative Nxf1 in A549 cells, with transfection efficiency ~70% as monitored by co-transfection with eGFP. We detect less virus production in cells inhibited for Nxf1-mediated nuclear export via expression of dominant negative Nxf1 than control (Figure 1A). 48 hours post transfection with DNA plasmids to express dominant negative Nxf1, A549 cells were infected with influenza A Udorn at MOI of 2.5 to assay single cycle infection. After 1-hour incubation with virus inoculum, cells were thoroughly washed and media samples collected. Collected media was used in plaque assay to calculate virus titer. Virus production was significantly inhibited at 12 hours post infection in A549 cells expressing the dominant negative Nxf1 protein (Figure 1A). These results are consistent with the notion that Nxf1 is required for optimal influenza production during infection [12].

To examine viral mRNA expression and export, A549 cells were fractionated at 3.5 hours post infection and proteins isolated from both the nuclear and cytoplasmic fractions. Western blot confirms cellular fractionation as SP1, a protein localized to the nucleus, is detected only in the nuclear fraction, and Hsp90, a protein localized to the cytoplasm, is detected only in the cytoplasmic fraction (Figure 1B). RNA was isolated from the cytoplasmic fraction and quantified using a nanospectrophotometer, and equal concentrations of RNA were subject to reverse transcription using oligo dT and gene specific PCR with primers to amplify influenza genes. We show results from three independent biological trials of RNA isolated from the cytoplasmic fraction using both semi-quantitative PCR, where product is taken over sequential cycles during exponential amplification and examined via ethidium bromide and gel electrophoresis, and triplicate quantitative PCR, where product is assessed by SYBR Green at each cycle and graphed relative to the control (uninhibited) sample. In all trials our data revealed that while NP mRNA expression in the cytoplasm was significantly decreased in A549 cells with Nxf1-mediated export inhibited, the polymerase encoding mRNAs, PA, PB1, and PB2 were not significantly affected (Figure 1C and D).

**Figure 2:**
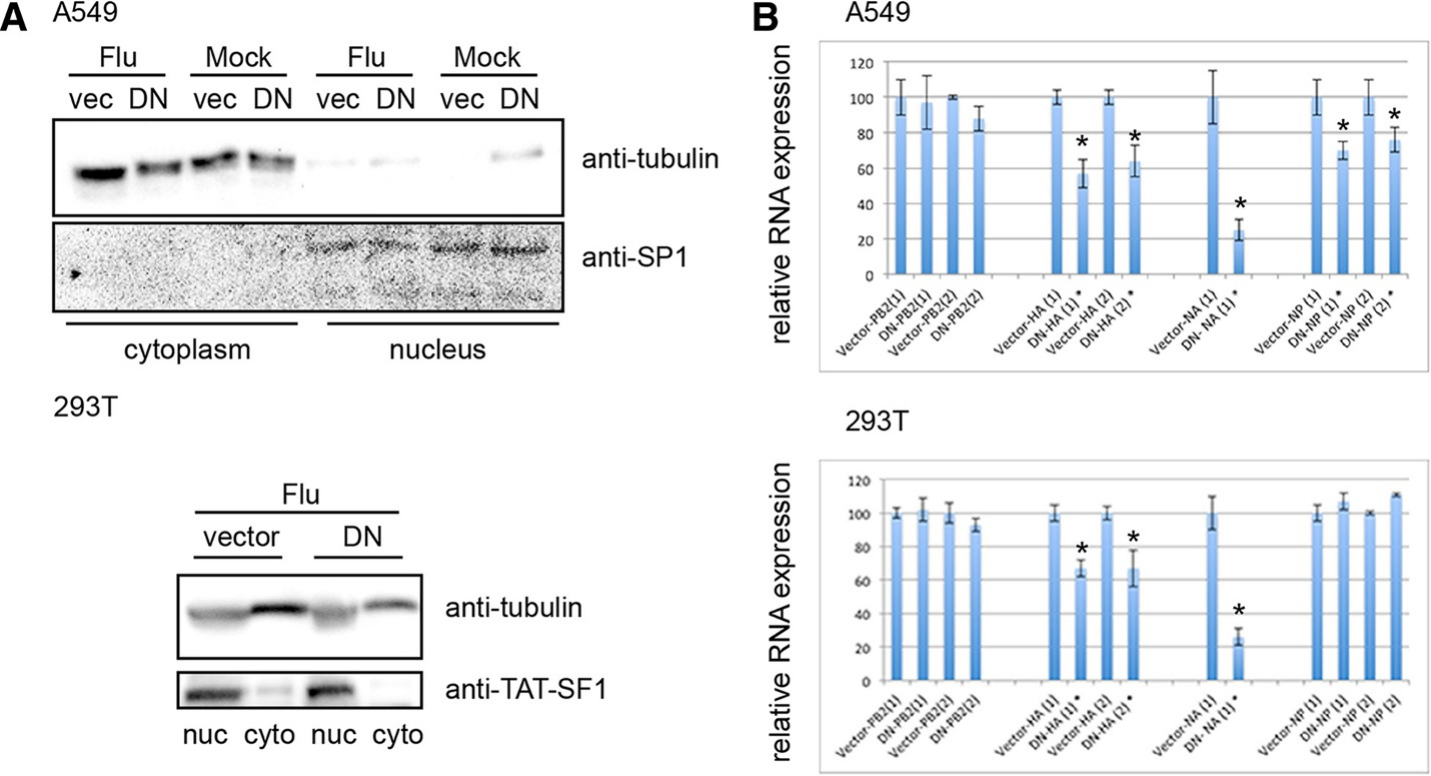
**NP mRNA analysis reveals a cell type difference in dependence of Nxf1-mediated nuclear export.** A549 and 293 T cells were transfected with plasmid to express dominant negative Nxf1 (DN) or vector control (vec), infected with influenza A Udorn at 2.5 MOI 48 hours post transfection, and fractionated 3.5 hours post infection. **A**. Cytoplasm and nuclear protein fractions were separated by SDS-PAGE and subject to Western blot to detect SP1, TAT-SF1, or tubulin. Shown is a representative blot from one biological trial. **B**. RT-qPCR of RNA isolated from the cytoplasm fraction. RNA was quantified and equal concentrations subject to RT with oligo dT. Gene specific PCR was performed using primers to amplify NP, PA, PB1 and PB2 as indicated. Data shown is from two biological independent trials of more than 5 repeats, each trial performed in triplicate PCR. Delta Ct was calculated to determine relative RNA expression. Raw CT values were analyzed in Microsoft Excel using 2^ΔCt(average control- average treated)^. Standard error was obtained by calculating the standard deviation of the sample set divided by the square root of the sample set size, and indicated using error bars. Significance was determined using a two-tailed T-Test conducted in Microsoft Excel, and judging any p value less than .05 as significant, indicated by an asterisk.

Our data is both similar and different to one published report [13], which concluded that early expressed mRNAs, NP and PB2, were less dependent on Nxf1-mediated nuclear export, with NP mRNA showing the least dependency. To understand the discrepancy regarding NP mRNA nuclear export, we addressed differences in the experimental design to determine what could account for the variance. In addition to different mechanism of inhibition, both the cell type and influenza strain used were different in our study. Therefore, we repeated our experimental approach in both 293 T and A549 cells, where we observed ~90% and ~70% transfection efficiency respectively, as monitored by co-transfection with eGFP. Western blot demonstrates decent cellular fractionation with tubulin, primarily in the cytoplasmic fraction and SP1 or TAT-SF1, nuclear localized proteins, detected in the nuclear fraction (Figure 2A). There is a background signal of unconfirmed identity, which migrates slightly below tubulin, detected in the 293 T nuclear fractions probed with anti-tubulin. We show triplicate RT-qPCR of isolated cytoplasmic RNA from two independent biological trials in each cell type to demonstrate that while NP mRNA nuclear export was Nxf1-mediated in A549 cells, NP mRNA nuclear export had no dependence on Nxf1 in 293 T cells (Figure 2B). Our studies reveal there is a cell type difference for influenza NP mRNA nuclear export. It is important to report and acknowledge cell type molecular differences to facilitate the transition from basic research to application. Further, our data support Nxf1-mediated nuclear export is functional during influenza infection and is important for optimal virus production.

### Immuno-purification of Nxf1 in A549 cells reveals NP, HA, and NA influenza viral mRNAs associate with Nxf1, while PA, PB1, and PB2 mRNAs do not

**Figure 3:**
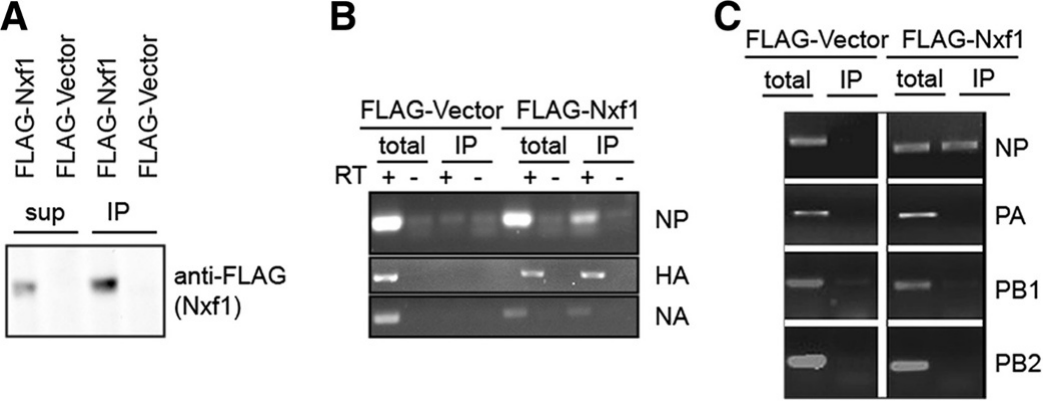
**Nxf1 is associated with NP, HA, and NA influenza mRNAs in A549 cells.** A549 cells were transfected with plasmid to express FLAG-Nxf1 or FLAG-Vector control and infected with influenza A Udorn at 2.5 MOI 48 hours post transfection. Cells were collected 7 hours post infection and total extract prepared and subject to immuno purification using anti-FLAG antibody coupled to beads. Protein and RNA were isolated from total extract, immuno purified beads, and supernatant. **A**. Protein from the immuno purified beads (IP) and supernatant (sup) were separated by SDS-PAGE and subject to Western blot with anti-FLAG antibody to detect FLAG-Nxf1. Shown is a representative blot from one biological trial. **B**-**C**. RNA isolated from IP and total samples was quantified and equal concentrations were subject to RT with oligo dT. Gene specific PCR was performed using primers to amplify NP, PA, PB1, PB2, HA, and NA for 25 cycles. Data shown is from two representative trials of more than 5 repeats. In all cases the volume of cDNA used in the PCR step was 1/10^th^ that used in the IP samples, with the exception of NP amplification in **B**, where equal cDNA was used for total and IP, and total samples are likely at saturation limit in end point PCR.

To further support the use of Nxf1 for influenza NP mRNA nuclear export but not polymerase encoding mRNAs in A549 cells, we immuno purified Nxf1 and examined associated RNAs. A549 cells were transfected with DNA plasmid to express functional FLAG-tagged human Nxf1 protein or FLAG-Vector control, expressing only the FLAG epitope [20]. Transfection efficiency was ~70% in A549 cells as monitored by co-transfection with eGFP. 48 hours post transfection cells were infected at MOI of 2.5 for analysis of single cycle infection. Cells were collected at 7 hours post infection and total protein extracts prepared. The extracts were subject to immuno affinity purification using anti-FLAG antibodies coupled to beads. Samples were analyzed by Western blot to confirm immuno purification of FLAG-Nxf1 (Figure 3A). While the Western blot results show not all expressed FLAG-Nxf1 was purified, as some FLAG-Nxf1 remains in the supernatant (SUP), sufficient amounts of FLAG-Nxf1 were present in the immuno purified (IP) sample for analysis of co-associated mRNAs. RNA was isolated from total extract and IP sample of infected cells expressing FLAG-Nxf1 or FLAG-vector negative control. RNA was analyzed with a nanospectrophotometer at absorbance 260 to calculate concentration. Equal concentrations of RNA from both the total and IP samples from both FLAG-Nxf1 and FLAG-vector negative control were subject to reverse transcription with oligo dT to generate cDNA of any associated polyadenylated RNAs. As expected, we routinely recovered less RNA in the FLAG-vector negative control IP compared to FLAG-Nxf1 IP. Therefore, to use equal RNA concentrations we consistently used a greater volume of sample recovered from the FLAG-vector negative control IP, which still showed little/no non-specific association with the highly expressed influenza mRNAs, strengthening the stringency of our experiment (Figure 3B and C). Furthermore, given that PCR is a very sensitive technique, which can amplify trace quantities of cDNA, we included a total control using 10% of cDNA from the total RNA sample; thus representing 10% of the amount processed in the IP sample. This provided an ~10% cut-off for interaction detection; if 10% of total cDNA showed product at PCR cycle 25 but the IP sample did not, we considered this no association with Nxf1, whereas if the IP sample did show product we considered this Nxf1-associated. Given Nxf1-mRNA is a dynamic interaction which only occurs during the act of mRNA nuclear export, ~10% of the mRNA showing interaction was reasoned to be an appropriate cut-off to avoid detection of false positives which could occur given the sensitivity of PCR. We find that while HA, NA, and NP mRNAs were specifically associated with FLAG-Nxf1; PA, PB1, and PB2 mRNAs were not (Figure 3B and C). Our results are both in agreement and disagreement with published studies [14], which conclude that NA, M1, and PB1 mRNAs associate with Nxf1 (also called TAP). Again there are many variables that could account for the discrepancy including virus subtype (Udorn vs PR8) and cell line utilized (A549 vs MDCK, Madin-Darby canine kidney). Our experiments demonstrate a specific and physical association of HA, NA, and NP influenza mRNAs with Nxf1 in A549 cells but no association with PA, PB1, and PB2 mRNAs. These immuno purification data are in agreement with our results obtained by inhibition of Nxf1. Together our data conclude that HA, NA, and NP mRNA nuclear export is Nxf1-mediated in A549 cells, while the polymerase encoding mRNAs, PA, PB1, and PB2, use an Nxf1-independent nuclear export mechanism.

### Influenza NS1 protein also associates with Nxf1/RNA immuno purified complexes

**Figure 4:**
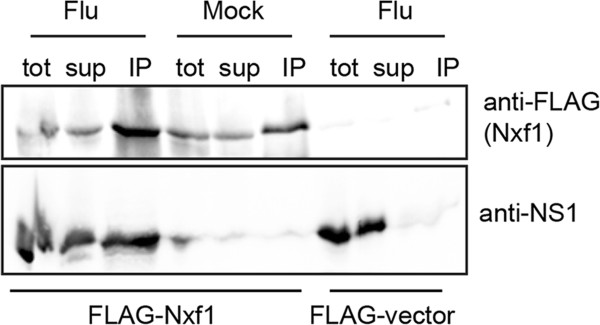
**Nxf1 is associated with influenza NS1 protein in A549 cells.** A549 cells were transfected with plasmid to express FLAG-Nxf1 or FLAG-Vector control and infected with influenza A Udorn or mock infected at 48 hours post transfection. Cells were collected 7 hours post infection and total extract was prepared and subject to immuno purification using anti-FLAG antibody coupled to beads. Proteins from the total extract (tot), immuno purified beads (IP), and supernatant (sup), were separated by SDS-PAGE and subject to Western blot with anti-FLAG antibody to detect FLAG-Nxf1 and anti-NS1 (Udorn) to detect influenza NS1 protein. Shown is a representative blot from one biological trial.

We confirm the reported influenza NS1 - Nxf1 protein interaction observed in kidney cells [14, 15] in A549 cells (Figure 4). This interaction is speculated to inhibit Nxf1 nuclear export as a mechanism to boost host shut off of antiviral gene expression [15] and enhance intron-less mRNA nuclear export [14]. It is possible NS1-Nxf1 protein interaction has a dual role; perhaps by facilitating and/or regulating select influenza mRNA nuclear export, host mRNA nuclear export is simultaneously inhibited. However, it is known from commonly utilized reconstituted vRNP assays that only influenza NP and RdRP (PA, PB1, PB2) proteins are required for mRNA production and subsequent protein expression from all vRNA templates, demonstrating NS1 is not required for efficient influenza mRNA nuclear export. On the other hand, NS1 has been speculated to play regulatory roles in influenza mRNA synthesis [21], splicing [22], nuclear export [23], and translation [24]. However, NS1 deletion viruses are viable in interferon (IFN) deficient Vero cells, underscoring the main function of influenza NS1 as an IFN antagonist [25]. In addition, host mRNA transcription is targeted for shut off by influenza at a minimum of two steps prior to mRNA nuclear export; loss of the 5’ cap structure on nascent mRNAs due to influenza ‘cap-snatching’ [5], and inhibition of host mRNA polyadenylation due to influenza NS1 interaction with host cleavage and polyadenylation specificity factor 30 kDa subunit (CPSF30) [26]. While it is unclear how much contribution to host shut off is due to NS1-Nxf1 interaction, viruses often employ multiple targets to counter the host antiviral defenses. While we do not yet completely understand the role of the NS1-Nxf1 protein interaction, it is clear that Nxf1-mediated nuclear export is functional during the influenza life cycle for nuclear export of at least some select influenza mRNAs and required for optimal influenza replication and virus production.

### Leptomycin B treatment inhibits virion production but not influenza PA, PB1, or PB2 mRNA nuclear export in A549 cells

**Figure 5:**
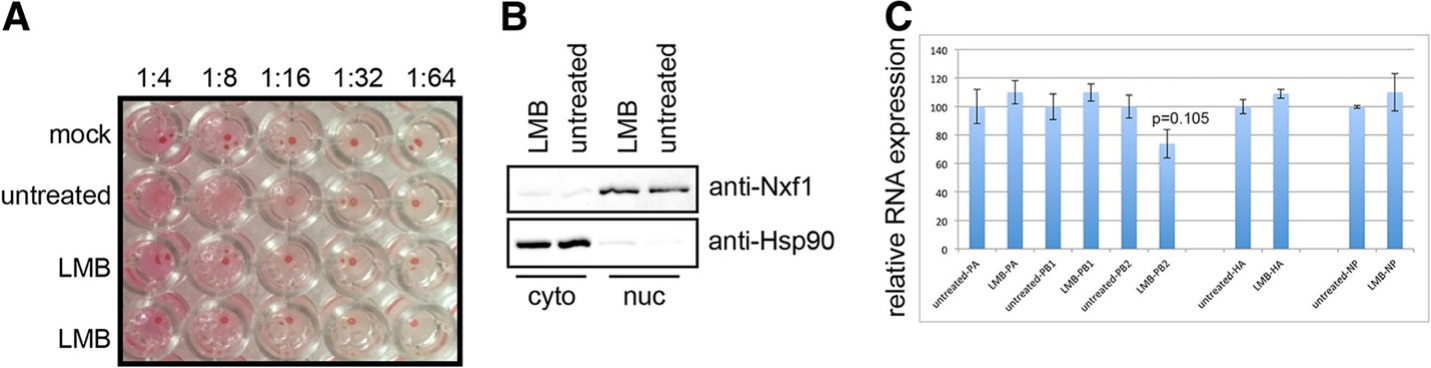
**Leptomycin B treatment does not alter cytoplasmic influenza PA, PB1, or PB2 mRNA in A549 cells.** A549 cells were infected with influenza A and virus allowed to adhere for 1 hour at which time virus inoculum was removed and replaced with media containing 10nM leptomycin B (LMB) to inhibit Crm1-mediate nuclear export or untreated media as control. **A**. Media samples from mock infected, LMB treated and untreated infected cells (top rows infected at MOI 1.4, bottom row infected at 2.8 MOI) were collected 36 hours post infection and subject to HA assay using two-fold dilutions. **B**. Infected LMB treated and untreated cells were fractionated at 3.5 hours post infection. Cytoplasm and nuclear protein fractions were separated by SDS-PAGE and subject to Western blot to detect Nxf1 and Hsp90. **C**. RT-qPCR of RNA isolated from the cytoplasm fraction 3.5 hours post infection. RNA was quantified and equal concentrations subject to RT with oligo dT. Gene specific PCR was performed using primers to amplify PA, PB1, PB2, HA, and NP as indicated. Data shown is from one biological trial performed in triplicate. Delta Ct was calculated to determine relative RNA expression. Raw CT values were analyzed in Microsoft Excel using 2^ΔCt(average control- average treated)^. Standard error was obtained by calculating the standard deviation of the sample set divided by the square root of the sample set size, and indicated using error bars. Significance was determined using a two-tailed T-Test conducted in Microsoft Excel, and judging any p value less than 0.05 as significant, no genes showed statistical difference in relative expression of RNA. PB2 p value was 0.105.

While published data suggests no influenza mRNAs utilize Crm1 for nuclear export [13, 14, 17, 18], these studies were carried out in kidney cell lines. Given the cell type difference regarding NP mRNA nuclear export and the lack of analysis of the role of Crm1 in a lung cell line, we readdressed this question in A549 cells. Consistent with the previous studies, we found no role for Crm1 in influenza mRNA nuclear export in A549 cells. After the initial virus attachment period, cells were treated with 10 nM LMB during viral infection at MOI of 1.4 or 2.8. To assess inhibition of Crm1 nuclear export, cells were washed well after 1 hour of incubation with virus inoculum and media samples taken. HA assay confirms no detectable virions are produced in cells treated with LMB in contrast to untreated cells, which produced virions detectable by HA (Figure 5A). This result suggests Crm1-mediated nuclear export is inhibited in the LMB treated samples because vRNP nuclear export during virion assembly requires Crm1-mediated nuclear export [16, 17]. To evaluate influenza mRNA nuclear export, cells were collected at 3.5 hours post infection, fractionated, and protein isolated from both nuclear and cytoplasmic fractions. Western blot confirms cellular fractionation as Nxf1, a protein localized to the nucleus, is detected only in the nuclear fraction, and Hsp90, a protein localized to the cytoplasm, is detected only in the cytoplasmic fraction (Figure 5B). RNA was isolated from the cytoplasmic fraction, quantified using a nanospectrophotometer, and equal concentrations were subject to reverse transcription using oligo dT and gene specific quantitative PCR in triplicate with primers to amplify influenza genes. We found no significant difference between LMB treated and untreated samples for cytoplasmic levels of influenza HA, NP, PA, PB1, and PB2 mRNAs (Figure 5C). However, cytoplasmic PB2 mRNA was ~25% decreased in LMB treated cells with a p-value of 0.105, which may represent some dependency on Crm1 nuclear export but was not deemed significant with a cut off of p < 0.05. Still, from this experiment we conclude that Crm1 is not utilized for intron-less influenza mRNA nuclear export in A549 cells. Together our results reveal that the influenza polymerase encoding mRNAs, PA, PB1, and likely PB2, use neither the major cellular mRNA nuclear export factor, Nxf1, nor the alternate Crm1 nuclear export factor for mRNA nuclear export in human lung adenocarcinoma epithelial cell line (A549).

## Conclusion

There are three main conclusions from our research results. The first is that Nxf1-mediated nuclear export is required for optimal influenza virus production in all cell types examined. When Nxf1-mediate nuclear export is inhibited by expression of dominant negative Nxf1 protein there is less nuclear export of select influenza mRNAs and less virus production. These data suggest that Nxf1-mediated nuclear export needs to be functional for optimal influenza replication. Further, select influenza mRNAs were found associated with Nxf1 in the cell, strongly supporting use of Nxf1-mediated nuclear export by these select viral mRNAs.

The second main conclusion is the recognition that model cell type can influence molecular mechanisms of influenza infection. We clearly demonstrate that NP mRNA nuclear export is Nxf1-mediated in A549 cells but Nxf1-independent in 293 T cells. This conclusion is important to properly assess research results for relevance to application. While 293 T cells are routinely used in research because of excellent ability to take up DNA, they are kidney cells and thus may not be the best human model cell line to study the molecular mechanism of the respiratory influenza A virus; A549 lung cells may represent a better model. Our study is a needed reminder that cell type can influence molecular mechanisms and this must be taken into consideration when analyzing, compiling, and comparing data.

The third and in our opinion most important main conclusion is that the polymerase encoding mRNAs, PA, PB1, and likely PB2, appear to use neither defined host mRNA nuclear export pathway in both cell types examined. This is an exciting result as it implies these viral mRNAs utilize an uncommon mRNA nuclear export pathway. While there are other RNA nuclear export factors, such as Exportin T to export tRNAs [27] and Exportin 5 to export pre-micro-RNAs [28], these factors have not as yet been implicated in export of mRNAs, viral or host. It may be that influenza has hijacked one of these non-coding RNA export pathways to export viral mRNAs. Further, Nxf1 is a member of a family of **n**uclear e**x**port **f**actors, and it could be that influenza polymerase encoding mRNAs have hijacked a less characterized member of this family. It is also possible that the influenza polymerase encoding mRNAs utilize an as yet unidentified and uncharacterized nuclear export pathway which likely function in atypical mRNA nuclear export pathway(s) and may represent feasible targets for future development of innovative antiviral therapies. Identification of the host factors that participate in the nuclear export of influenza PA, PB1, and PB2 mRNAs is a goal of our future research.

## Methods

### Cells, virus, plasmids, LMB, and antibodies

293 T human embryonic kidney cell line, A549 human lung adenocarcinoma epithelial cell line, and MDCK Madin-Darby canine kidney cell line were purchased from ATCC American tissue culture collection and maintained at 37°C with 5% CO2 in DMEM with 10% FBS. Influenza A/Udorn/307/1972(H3N2) was generated from 12 plasmids using reverse genetics as described [29]. The plasmids required for reverse genetics were kindly provided by R. Krug. Plasmids encoding dominant negative DN - Nxf1 (TapA17) was kindly provided by B. Cullen [19] and plasmid encoding FLAG-Nxf1 was kindly provided by J. Steitz [20]. Plasmid DNA was purified using QIAGEN maxi or mini prep kit per manufacturers protocol. Leptomycin B (LMB) was purchased from Fisher. α-Tubulin, α-SP1, α-HSP90, α-NXF1, and α-FLAG were purchased from Abcam and used per manufacturers instructions. α-NS1 Udorn was kindly provided by R. Krug. Secondary HRP coupled α-Mouse and α-Rabbit were purchased from Pierce and used per manufacturer’s instructions.

### Transfection

293 T or A549 cells were grown to approximately 70% confluency in 100 mm dishes or 6-well plates depending on experiment.

pcDNA plasmids encoding FLAG-NXF1 (10 μg) and FLAG-Vector (9 μg) with eGFP (1 μg), or DN-Nxf1 (10 μg) and CMV (9 μg) with eGFP (1 μg) were transfected into cells (100 mm dish) using Mirus transfection DNA to reagent at a ratio of 1:3 (293 T cells) or 1:2 (A549 cells). eGFP was used to monitor transfection efficiency, which was ~70% in A549 cells and ~90% in 293 T cells. 48 hrs post transfection one set was mock infected and the other infected at MOI of 2.5.

### LMB treatment

Cells were allowed to incubate with viral inoculum (MOI 1.4 or 2.8) for 1 hour; after attachment viral inoculum was removed and 10nM LMB was added along with the incubation media in treated samples for the remainder of influenza infection period.

### Influenza A infection

Cells were infected with MOI of 1.4, 2.5, or 2.8 as indicated. Cells were first washed with PBS and overlaid with viral inoculum for 1 hour with gentle shaking every 15 minutes to ensure cells did not dry out and virus attachment occurred. After 1 hour virus inoculum was removed and replaced with media containing 2.5% FBS. For experiments where virion or virus production was assessed, cells were washed numerous times prior to addition and sampling of media. Furthermore, a control sample was taken at this point to ensure minimal/no virus left behind from inoculum. For examination of mRNA nuclear export under conditions of Nxf1 or Crm1 inhibition, cells were collected at 3.5 hours post infection. For examination of direct Nxf1-mRNA interaction, cells were collected at 7 hours post infection.

### Plaque and HA assays

Serial dilutions of media samples were subject to standard plaque or HA assay. For plaque assay, media from triplicate samples were diluted and used to infect confluent MDCK cells. Titers from triplicate trials were averaged and standard error was obtained by calculating the standard deviation of the sample set divided by the square root of the sample set size, and indicated using error bars. Significance was determined using a two-tailed T-Test conducted in Microsoft Excel, and judging any p value less than .05 as significant. For HA assay two-fold dilutions of media was mixed with chicken red blood cells.

### Cellular fractionation and isolation of protein and RNA

At 3.5 hours post infection, cells were pelleted by centrifugation and cellular pellets were washed in 5X volume of cell pellet with Reticulate Standard Buffer (RSB: 10 mM Tris HCl pH7.5, 10 mM KCl, 1.5 mM MgCl_2_) containing protease and RNase inhibitors. Cells were then re-suspended in RSB at 10X the volume of the cell pellet and incubated on ice for 10 minutes. NP-40 was added to a final concentration of 0.2% to disrupt plasma membranes. Visual inspection of the cells before and after addition of NP-40 ensured burst plasma membranes and intact nuclei. Nuclei were pelleted by centrifugation at 300×g for 8 minutes at 4°C. The cytoplasmic extract was collected and the nuclear pellet was re-suspended in Dignam Buffer C without glycerol (20 mM HEPES pH 7.9, 0.42 M NaCl, 1.5 mM MgCl_2_, 0.2 mM EDTA) and containing protease and RNase inhibitors, to release nuclear molecules. Both nuclear and cytoplasmic extracts were clarified from debris by high-speed centrifugation for 10 minutes at 4°C. An equal amount of 20 mM HEPES pH 7.9, 0.2 mM EDTA was added to the nuclear extract to reduce the total NaCl and MgCl_2_ concentrations. This was used as cytoplasmic and nuclear protein extracts.

To isolate RNA, equal volume of Phenol/Chloroform/Isoamyl alcohol (25:24:1) was added to a portion of the cytoplasmic and nuclear fractions. Samples were vortexed 4 times for 10 seconds and placed on ice in between. Samples were centrifuged at 13,000 RPM for 10 minutes at 4°C. Aqueous layer was collected and 0.5 volume NH_4_OAc (7.5 M) and 2X volume 100% EtOH was added and RNA was allowed to precipitate overnight at -80°C. Samples were centrifuged at 13,000 RPM for 20 minutes at 4°C. Pellet was washed in 75% EtOH and centrifuged at 13,000 RPM for 5 minutes at 4°C. EtOH was removed and pellet was allowed to air dry for 10 minutes and resuspended in 10 mM Tris in DEPC H_2_O; amount dependent on size of RNA pellet. RNA was quantified using a nanospectrophotometer and absorbance at 260.

### Total extract preparation for immunopurification and associated RNA isolation

For immuno purification experiments, cell pellets were resuspended in 1 mL Sonication Buffer (100 mM Tris HCl pH 7.5, 100 mM NaCl, 2.5 mM MgCl_2_, 0.5% Triton ×-100) containing protease and RNase inhibitors. Cells were lysed using Fisher Scientific Sonic Dismembrator for 30 pulses at 30%, output 3–4. Sonicated materials were loaded onto a 30% sucrose cushion (30% Sucrose, 10 mM Tris HCl pH 7.5, 100 mM NaCl, 2.5 mM MgCl_2_) and centrifuged at 4000 RPM for 15 minutes at 4°C to clarify total protein extract.

#### Immunopurification

A portion of total protein extract was incubated with α-FLAG antibody (Stratagene) (1:50) and protease inhibitors for 1 hour at 4°C. Extracts were then incubated with PA/G sepharose beads washed with sonication buffer and containing protease inhibitors, at 4°C overnight. The samples were then spun for 8 seconds at 13,000 RPM at 4°C. Supernatant was collected. Beads were washed 3 times in 1 mL sonication buffer with RNase inhibitors. With each wash samples were then centrifuged for 8 seconds at 13,000 RPM at 4°C. 1/3 of the immuno purification from last wash was saved for protein analysis, and 2/3 was saved for RNA isolation.

#### RNA isolation

Samples were first subject to protease degradation by incubation with protease K and equal volume of Phenol/Chloroform/Isoamyl alcohol (25:24:1) was added to the resuspended bead immunopurification sample and total extract and samples processed as previously described above.

### SDS-PAGE and Western Blot

Protein extracts were separated by SDS-10% PAGE. Proteins were transferred to nitrocellulose using Fisher semi-dry blot apparatus and probed with primary and HRP-conjugated secondary antibodies as indicated. Pierce ECL reagents were used to detect HRP conjugated secondary antibody. Blots were developed using the Chemi-Hi setting on the ChemiDoc™ XRS (BioRad) system and digital images were obtained using Quantity One software. Digital images were exported as raw data TIFF files and image prepared using Adobe Photoshop to crop photos and adjust exposure, and Adobe Illustrator to add figure text labels.

### Reverse transcription – PCR

RNA was first quantified by spectrophotometry and separated on a 1% bleach/1% agarose gel to observe rRNA in total and cytoplasmic RNA preparations. For inhibition experiments, 1 μg cytoplasmic RNA was subject to reverse transcription using Promega AMV reverse transcription system per manufacturer’s protocol with oligo dT as primer. For immuno purification experiments, 150-500 ng RNA was subject to reverse transcription depending on RNA recovery; however in all cases equivalent RNA concentration was used for all samples in the RT step.

#### End point and semi-quantitative PCR

For analysis of mRNAs associated with Nxf1, 10% cDNA from total samples relative to 100% cDNA from IP was used for PCR (for example 1ul total and 10ul IP). For analysis of mRNA in cells inhibited for Nxf1-mediate nuclear export, equal volume cDNA was subject to gene specific PCR. For analysis of mRNAs associated with Nxf1 samples were taken at cycle 25. For analysis of mRNA in cells inhibited for Nxf1-mediate nuclear export samples were taken at sequential cycles as indicated to confirm analysis within the PCR exponential amplification curve. Samples were observed by ethidium bromide 1% agarose gel electrophoresis.

Primers for end point and semi-quantitative PCR:

NP (forward - CCAGAAGAAGTGTCCTTCCG,

reverse - CGTACTCCTCTGCATTGTCTCC),

PB1 (forward - CCCCTGAATCCATTTGTCAGCCATA,

reverse - ATGAAGGACAAGCTAAATTG),

HA (forward - GCTCTGGAGAACCAACATACAA,

reverse - ACAAGGGTGTTTTTAATTACTAATA),

PB2 (forward - CCACCCAGATAATAAAGCTTCTCCCC,

reverse - GTCAGTAAGTATGCTAGAGTCCCG),

PA (forward - ATGACCAAAGAGTTTTTTGAGAATA,

reverse - GTATGGATAGCAAATAGTAGCATTG).

#### Quantitative PCR

Equal amounts of cDNA were used in triplicate reactions. Quantitative PCR reactions were run in the Applied Biosystems StepOnePlus Real Time PCR system using SYBR Green Master mix (Applied Biosystems), with ROX as the reference dye.

Primers for gene specific qPCR:

NA (forward TGTGTGCTCAGGGCTTGTTG,

reverse CTCCGTGATTCCCTTTCTCATT)

HA (forward ACTGAAGTCAGGATACAAAGACTGGAT,

reverse CCCCAGCAAAACAACACAAA)

NP (forward GTGTGCAACCTGCATTTTCTGT,

reverse TCTGAGGTTCTTCCCTCCGTATT)

PA (forward GGACAAATGGAACATCAAAGATTAAA

Reverse CAGAAGACTCGGCTTCAATCATG)

PB1 (forward GGGAAAGGATACATGAACGAAAGT

reverse ACTTTAGGTCAATGCTTGCTAGCA)

PB2 (forward AATAAAGCTTCTCCCCTTTGCA

reverse CCCTCACATTCACAGTCAATGAA)

#### PCR cycle

PCR was performed in a standard 3 cycle PCR with denaturation at 95°C for 30 seconds, annealing temperature of 55°C for 30 seconds, and extension temperature of 72°C for 30 seconds.

#### Statistical Analysis of qPCR Data

Raw CT values were analyzed in Microsoft Excel using the 2^ΔCt(control-treated)^ formula of 2^^CTaverage Control sample^/2^ ^CTaverage Treated sample^. Due to the robust host shut off that occurs during influenza infection, we were unable to reliably detect a reference gene but rather normalized to total RNA concentration. RNA OD to calculate concentration was taken in duplicate or triplicate and RNA analyzed using gel electrophoresis prior to reverse transcription to ensure equal rRNA concentration and no RNA degradation. Reverse transcription reactions were aliquot from a master mix to ensure all samples obtained equivalent AMV-RT enzyme. Standard error was obtained by calculating the standard deviation of the sample set divided by the square root of the sample set size, and indicated using error bars. Significance was determined using a two-tailed T-Test conducted in Microsoft Excel, and judging any p value less than .05 as significant.

## Authors’ information

SL and SB were former Master’s graduate students. SL is currently a medical student at St. George’s University, Grenada and SB is currently a medical student at Western University of Health Sciences, California. VP was a former MARC (Minority Access to Research Careers) undergraduate scholar and is currently a high school science teacher for San Bernardino City Unified School District, California. AM was a recent post baccalaureate student researcher. HMR was a former McNair undergraduate scholar who is currently a medical doctor in residence at the University of Southern California. All student researchers performed this work in the CSUSB laboratory of LLN, the PI and corresponding author.

## References

[1] **Centers for Disease Control** [http://www.cdc.gov/flu/]

[2] Lagacé-Wiens PRS, Rubinstein E, Gumel A (2010). Influenza epidemiology – past, present, and future. Crit Care Med.

[3] Russell CA, Fonville JM, Brown AE, Burke DF, Smith DL, James SL, Herfst S, van Boheemen S, Linster M, Schrauwen EJ, Katzelnick L, Mosterín A, Kuiken T, Maher E, Neumann G, Osterhaus AD, Kawaoka Y, Fouchier RA, Smith DJ (2012). The potential for respiratory droplet-transmissible A/H5N1 influenza virus to evolve in a mammalian host. Science.

[4] Herfst S, Schrauwen EJ, Linster M, Chutinimitkul S, de Wit E, Munster VJ, Sorrell EM, Bestebroer TM, Burke DF, Smith DJ, Rimmelzwaan GF, Osterhaus AD, Fouchier RA (2012). Airborne transmission of influenza A/H5N1 virus between ferrets. Science.

[5] Plotch SJ, Bouloy M, Ulmanen I, Krug RM (1981). A unique cap(m7GpppXm)-dependent influenza virion endonuclease cleaves capped RNAs to generate the primers that initiate viral RNA transcription. Cell.

[6] Poon LL, Pritlove DC, Fodor E, Brownlee GG (1999). Direct evidence that the poly (A) tail of influenza A virus mRNA is synthesized by reiterative copying of a U track in the virion RNA template. J Virol.

[7] Malim MH, Hauber J, Le SY, Maizel JV, Cullen BR (1989). The HIV-1 rev trans-activator acts through a structured target sequence to activate nuclear export of unspliced viral mRNA. Nature.

[8] Fornerod M, Ohno M, Yoshida M, Mattaj IW (1997). CRM1 is an export receptor for leucine-rich nuclear export signals. Cell.

[9] Gruter P, Tabernero C, von Kobbe C, Schmitt C, Saavedra C, Bachi A, Wilm M, Felber BK, Izaurralde E (1998). TAP, the human homolog of Mex67p, mediates CTE-dependent RNA export from the nucleus. Mol Cell.

[10] Pasquinelli AE, Ernst RK, Lund E, Grimm C, Zapp ML, Rekosh D, Hammarskjold ML, Dahlberg JE (1997). The constitutive transport element (CTE) of Mason-Pfizer monkey virus (MPMV) accesses a cellular mRNA export pathway. EMBO J.

[11] Bogerd HP, Echarri A, Ross TM, Cullen BR (1998). Inhibition of human immunodeficiency virus Rev and human T-cell leukemia virus Rex function, but not Mason-Pfizer monkey virus constitutive transport element activity, by a mutant human nucleoporin targeted to Crm1. J Virol.

[12] Hao L, Sakurai A, Watanabe T, Sorensen E, Nidom CA, Newton MA, Ahlquist P, Kawaoka Y (2008). Drosophila RNAi screen identifies host genes important for influenza virus replication. Nature.

[13] Read EK, Digard P (2010). Individual influenza A virus mRNAs show differential dependence on cellular NXF1/TAP for their nuclear export. J Gen Virol.

[14] Wang W, Cui ZQ, Han H, Zhang ZP, Wei HP, Zhou YF, Chen Z, Zhang XE (2008). Imaging and characterizing influenza A virus mRNA transport in living cells. Nucleic Acids Res.

[15] Satterly N, Tsai PL, van Deursen J, Nussenzveig DR, Wang Y, Faria PA, Levay A, Levy DE, Fontoura BM (2007). Influenza virus targets the mRNA export machinery and the nuclear pore complex. Proc Natl Acad Sci U S A.

[16] Neumann G, Hughes MT, Kawaoka Y (2000). Influenza A virus NS2 protein mediates vRNP nuclear export through NES-independent interaction with hCRM1. EMBO J.

[17] Elton D, Simpson-Holley M, Archer K, Medcalf L, Hallam R, McCauley J, Digard P (2001). Interaction of the influenza virus nucleoprotein with the cellular CRM1-mediated nuclear export pathway. J Virol.

[18] Amorim MJ1, Read EK, Dalton RM, Medcalf L, Digard P (2007). Nuclear export of influenza A virus mRNAs requires ongoing RNA polymerase II activity. Traffic.

[19] Kang Y, Cullen BR (1999). The human Tap protein is a nuclear mRNA export factor that contains novel RNA-binding and nucleocytoplasmic transport sequences. Genes Dev.

[20] Huang Y, Steitz JA (2001). Splicing factors SRp20 and 9G8 promote the nucleocytoplasmic export of mRNA. Mol Cell.

[21] Min JY, Li S, Sen GC, Krug RM (2007). A site on the influenza A virus NS1 protein mediates both inhibition of PKR activation and temporal regulation of viral RNA synthesis. Virology.

[22] Robb NC, Fodor E (2012). The accumulation of influenza A virus segment 7 spliced mRNAs is regulated by the NS1 protein. J Gen Virol.

[23] Schneider J, Wolff T (2009). Nuclear functions of the influenza A and B viruses NS1 proteins: do they play a role in viral mRNA export?. Vaccine.

[24] de la Luna S, Fortes P, Beloso A, Ortín J (1995). Influenza virus NS1 protein enhances the rate of translation initiation of viral mRNAs. J Virol.

[25] García-Sastre A, Egorov A, Matassov D, Brandt S, Levy DE, Durbin JE, Palese P, Muster T (1998). Influenza A virus lacking the NS1 gene replicates in interferon-deficient systems. Virology.

[26] Nemeroff ME, Barabino SM, Li Y, Keller W, Krug RM (1998). Influenza virus NS1 protein interacts with the cellular 30 kDa subunit of CPSF and inhibits 3’end formation of cellular pre-mRNAs. Mol Cell.

[27] Kutay U, Lipowsky G, Izaurralde E, Bischoff FR, Schwarzmaier P, Hartmann E, Görlich D (1998). Identification of a tRNA-specific nuclear export receptor. Mol Cell.

[28] Lund E, Güttinger S, Calado A, Dahlberg JE, Kutay U (2004). Nuclear export of microRNA precursors. Science.

[29] Neumann G, Watanabe T, Ito H, Watanabe S, Goto H, Gao P, Hughes M, Perez DR, Donis R, Hoffmann E, Hobom G, Kawaoka Y (1999). Generation of influenza A viruses entirely from cloned cDNAs. Proc Natl Acad Sci U S A.

